# Global trends in Alzheimer’s disease randomized controlled trials: a bibliometric analysis

**DOI:** 10.1590/1980-5764-DN-2025-0423

**Published:** 2026-06-15

**Authors:** Sanam Hosseinpoor-Dashatani, Narges Ebrahimi

**Affiliations:** 1 Tabriz University of Medical Sciences, Faculty of Health, Department of Epidemiology and Biostatistics, Tabriz, Iran.; 2 Tehran University of Medical Sciences, School of Public Health, Department of Health Education and Promotion, Tehran, Iran.

**Keywords:** Bibliometrics, Publications, Publishing, Alzheimer Disease, Randomized Controlled Trial., Bibliometria, Publicações, Editoração, Doença de Alzheimer, Ensaio Clínico Controlado Aleatório.

## Abstract

Alzheimer’s disease (AD) is the most common form of dementia worldwide, creating substantial clinical and socioeconomic burdens. Randomized controlled trials (RCTs) provide the highest level of evidence to evaluate interventions, yet global publication trends and thematic evolution have not been systematically analyzed.

**Objective:**

As far as we are aware, there has been no bibliometric analysis that has thoroughly assessed RCTs in AD, despite their pivotal influence on the development of treatment and prevention strategies. Therefore, in this study, we conducted a bibliometric mapping analysis of global RCTs on AD.

**Methods:**

A bibliometric analysis of human RCTs on AD from September 2010 to September 2025 was conducted using PubMed and Web of Science. VOSviewer was employed for keyword co-occurrence, co-authorship mapping, and co-citation analyses to identify research themes, collaborations, and temporal trends.

**Results:**

A total of 4,482 RCTs were identified, revealing five main themes: pharmacological interventions, lifestyle and prevention strategies, pathophysiological mechanisms, cognitive and behavioral interventions, and clinical trial methodology. After 2015, focus shifted from traditional pharmacology to multidomain, prevention-oriented, and precision-driven approaches. Emerging topics included digital health, gut microbiome, and machine learning. Collaboration networks highlighted the dominance of the US and Europe, with rapid growth in Asia and emerging regions.

**Conclusion:**

Findings indicate a paradigm shift in AD RCTs toward integrative, technology-enabled designs, emphasizing both pharmacological and non-pharmacological strategies. These trends can guide future global research priorities and intervention development.

## RESUMO

A doença de Alzheimer é a forma mais comum de demência em todo o mundo, criando encargos clínicos e socioeconômicos substanciais. Os ensaios clínicos randomizados fornecem o mais alto nível de evidência para avaliar intervenções, mas as tendências globais de publicação e a evolução temática não foram analisadas sistematicamente.

**Objetivo:**

Tanto quanto sabemos, não houve nenhuma análise bibliométrica que avaliasse exaustivamente os ensaios clínicos randomizados em doença de Alzheimer, apesar de sua influência fundamental no desenvolvimento de estratégias de tratamento e prevenção. Portanto, neste estudo, realizamos uma análise bibliométrica de mapeamento de ensaios clínicos randomizados globais sobre doença de Alzheimer.

**Métodos:**

Foi realizada uma análise bibliométrica de ensaios clínicos randomizados em humanos sobre doença de Alzheimer de setembro de 2010 a setembro de 2025 usando PubMed e Web of Science. O VOSviewer foi empregado para coocorrência de palavras-chave, mapeamento de coautoria e análises de cocitação para identificar temas de pesquisa, colaborações e tendências temporais.

**Resultados:**

Foram identificados 4.482 ensaios clínicos randomizados, revelando cinco temas principais: intervenções farmacológicas, estilo de vida e estratégias de prevenção, mecanismos fisiopatológicos, intervenções cognitivas e comportamentais e metodologia de ensaios clínicos. Após 2015, o foco mudou da farmacologia tradicional para abordagens multidomínios, orientadas para a prevenção e baseadas na precisão. Os tópicos emergentes incluíram saúde digital, microbioma intestinal e aprendizagem automática. As redes de colaboração destacaram o domínio dos Estados Unidos e da Europa, com rápido crescimento na Ásia e em regiões emergentes.

**Conclusão:**

Os resultados indicam uma mudança de paradigma nos ensaios clínicos randomizados de doença de Alzheimer em direção a projetos integrativos e habilitados por tecnologia, enfatizando estratégias farmacológicas e não farmacológicas. Essas tendências podem orientar as prioridades futuras de pesquisa global e o desenvolvimento de intervenções.

## INTRODUCTION

Alzheimer’s disease (AD) is the most common form of dementia. Current estimates indicate that more than 55 million people are living with dementia globally. Each year, nearly 10 million new cases are diagnosed, with AD playing a major role^1^. Beyond the devastating impact of AD on individuals and caregivers, it places an immense economic burden on healthcare systems. The worldwide costs of dementia, of which Alzheimer’s disease accounts for the majority, have surpassed USD 1 trillion annually^2^.

Randomized controlled trials (RCTs) are the cornerstone for assessing the efficacy and safety of pharmacological and non-pharmacological interventions in diseases. Their methodological rigor enables the achievement of the highest level of evidence to guide clinical practice and therapeutic development^3^. Early studies evaluated therapies aimed at symptom relief, including cholinesterase inhibitors and memantine. In contrast, contemporary RCTs have shifted toward modifying strategies, emphasizing monoclonal antibodies against amyloid and tau proteins^4,5,6^. In addition, lifestyle-based multidomain approaches, such as finger, have expanded the scope of AD research by addressing modifiable risk factors^7,8^. This diversification of therapeutic approaches reflects the underlying complexity of AD biology and highlights the need for multifactorial intervention strategies.

While multiple systematic reviews and meta-analyses have synthesized data from RCTs in AD, few studies have been devoted to examining the field through bibliometric methods. Bibliometric mapping offers a quantitative overview of publication trends, influential authors, citation impact, and emerging themes that are not captured^9,10,11,12,13,14^.

As far as we are aware, no bibliometric analysis has thoroughly assessed RCTs in AD, despite their pivotal influence on the development of treatment and prevention strategies. Therefore, in this study, we conducted a bibliometric mapping analysis of global RCTs on AD.

This study aimed to analyze global research trends in AD RCTs by conducting a comprehensive bibliometric mapping of publication patterns, research themes, and international collaborations.

## METHODS

### Study design

This study provides an extensive bibliometric analysis of RCTs in AD. The primary objectives was to identify publication patterns, determine the most influential authors, and highlight emerging thematic research fields. A two-step data collection strategy was employed to ensure comprehensive and accurate coverage of peer-reviewed RCTs, with subsequent analyses performed in VOSviewer.

### Search strategy

We employed a two-stage search strategy to systematically identify RCTs in AD. First, PubMed was searched using AD keywords {(“Alzheimer Disease”[MeSH Terms] OR “Alzheimer*”[tiab] OR “Alzheimer’s disease”[tiab] OR dementia[tiab] OR “cognitive decline”[tiab])}, restricting to human RCTs’ publications dated September 6, 2010, to September 6, 2025, using the PubMed Publication Date filter. All retrieved records were screened for the presence of a Digital Object Identifier (DOI). A total of 52 records lacked a DOI; these were excluded because DOI-based retrieval was required for metadata export into Web of Science (WoS) and subsequent bibliometric analysis. The final dataset used for bibliometric and network analyses consisted of 4,482 RCT-related records. Subsequently, the collected DOIs were imported into WoS to extract full metadata, including author affiliations, references, citation metrics, and abstracts. By employing this two-step approach, we maximized dataset coverage and the depth of metadata. PubMed facilitated comprehensive identification of relevant RCTs, whereas WoS provided enriched data suitable for advanced bibliometric and network analyses. 

Records were formatted for VOSviewer, enabling a comprehensive analysis that provides robust visualizations of trend patterns in AD RCT research.

### Cluster analysis and keyword processing

Bibliometric network and cluster analyses were conducted using VOSviewer. Prior to analysis, all author keywords and Keywords Plus were merged into a single keyword dataset. We performed standard harmonization procedures, including:

- merging synonyms and spelling variations (e.g., “amyloid β”, “amyloid-beta”, “Aβ” consolidated as “amyloid beta”),
- normalizing singular/plural forms,
- retaining author-derived terms that appeared in the title/abstract only if they occurred at least twice across the dataset after harmonization.

Also, two authors independently reviewed 10 representative articles per cluster (selected based on within-cluster relevance and citation strength) to confirm semantic coherence. Cluster labels were finalized by consensus and reflect the dominant conceptual themes (e.g., pharmacological interventions, lifestyle/prevention studies, mechanisms of disease, behavioral interventions, and trial methodology).

### Data analysis

We used VOSviewer to generate bibliometric networks. Co-authorship analyses at both author and country levels, keywords co-occurrence mapping, and co-citation by author and country were conducted. These network-based findings provided an integrated perspective on the field.

## RESULTS

### Descriptive overview

From PubMed, a total of 353,179 records were initially retrieved. After restricting the time frame to September 6, 2010, through September 6, 2025, the dataset was narrowed to 247,179 publications. When excluding non-human studies, 167,749 articles remained. Applying the PubMed filter for RCTs identified a total of 4,534 eligible records.

Subsequently, 52 publications that lacked DOIs were excluded. The final set of 4,482 RCTs was exported and imported into the WoS database. This step enabled the retrieval of comprehensive bibliographic and citation information, which formed the basis for the bibliometric mapping analysis.

### Keyword co-occurrence

The bibliometric search yielded 695 distinct keywords, highlighting the heterogeneous and multidisciplinary nature of this research field.

**Figure 1:**
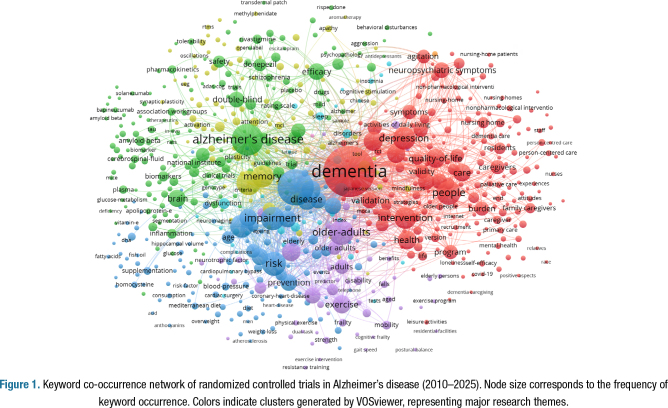
Keyword co-occurrence network of randomized controlled trials in Alzheimer’s disease (2010–2025). Node size corresponds to the frequency of keyword occurrence. Colors indicate clusters generated by VOSviewer, representing major research themes.

Through cluster analysis, five core research themes were highlighted, which are depicted in the network visualization (Figure 1):

1. Drug interventions (Aβ, acetylcholine, acetylcholinesterase inhibitors),
2. Lifestyle and prevention (exercise, diet, cognitive training),
3. Pathophysiological mechanisms (biomarkers, tau, inflammation),
4. Cognitive and behavioral interventions (memory training, depression, cognition), and
5. Clinical trial methodology (placebo, double-blind, multicenter design).

**Figure 2:**
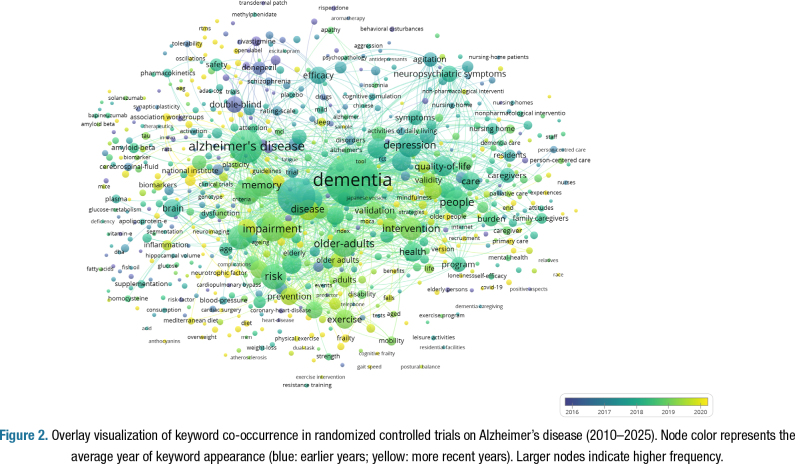
Overlay visualization of keyword co-occurrence in randomized controlled trials on Alzheimer’s disease (2010–2025). Node color represents the average year of keyword appearance (blue: earlier years; yellow: more recent years). Larger nodes indicate higher frequency.

Analysis of temporal trends revealed a clear progression. Studies from 2010 to 2015 primarily centered on traditional pharmacological targets, including *A*β*, acetylcholinesterase, and memantine*. In contrast, studies published after 2015 increasingly shifted toward prevention-oriented domains, highlighting lifestyle modification, multimodal therapeutic designs, and personalized approaches. Also, emerging themes involved digital health, the gut microbiome, and machine learning applications. These are depicted in the network visualization (Figure 2).

### Co-authorship by countries

In the field of AD RCTs, the United States ranked the highest productivity and impact. Research clusters in Europe and North America, including England, Germany, France, Canada, and Sweden, consistently exhibited high productivity and significant scholarly impact. In Asia, countries such as China, Japan, South Korea, and Singapore have experienced substantial growth in recent years. Singapore demonstrates high impact, averaging more than 90 citations per paper and a normalized citation impact of 3.7.

**Figure 3:**
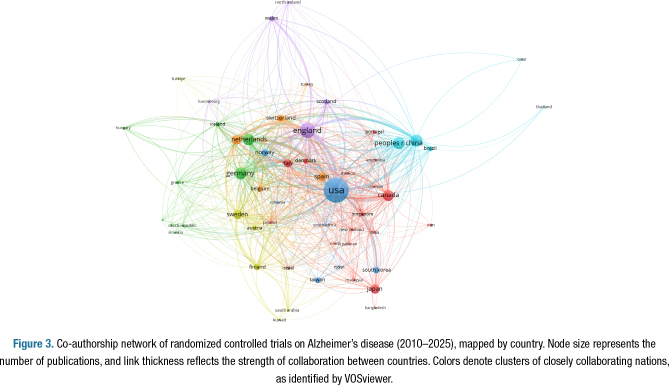
Co-authorship network of randomized controlled trials on Alzheimer’s disease (2010–2025), mapped by country. Node size represents the number of publications, and link thickness reflects the strength of collaboration between countries. Colors denote clusters of closely collaborating nations, as identified by VOSviewer.

**Figure 4:**
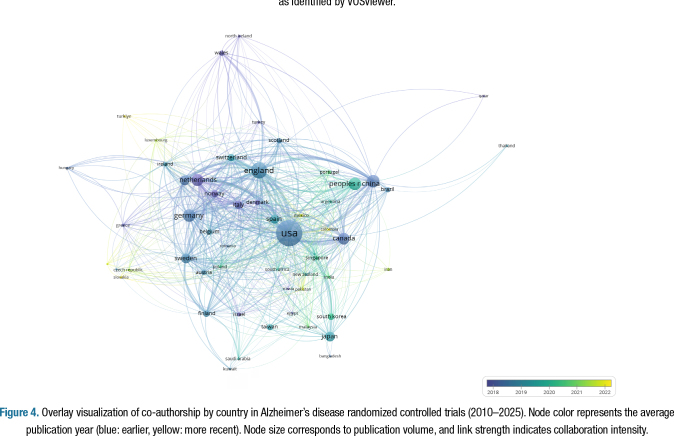
Overlay visualization of co-authorship by country in Alzheimer’s disease randomized controlled trials (2010–2025). Node color represents the average publication year (blue: earlier, yellow: more recent). Node size corresponds to publication volume, and link strength indicates collaboration intensity.

Co-authorship network analysis identified four major clusters of international collaboration (Figures 3 and 4):

- Cluster 1 (North America): The United States and Canada dominate this cluster, representing the primary force in Alzheimer’s RCT research.
- Cluster 2 (Europe): Comprising Germany, France, Sweden, Finland, the Netherlands, and Italy, this cluster is particularly strong in lifestyle interventions, exemplified by initiatives such as the FINGER trial.
- Cluster 3 (Asia–Oceania): Including China, Japan, and Australia, the focus of this cluster is primarily on novel pharmacological therapies and genetic studies.
- Cluster 4 (Emerging Countries): Countries such as Iran, India, Turkey, and Brazil demonstrate fewer publications; however, the average publication year post-2020 indicates rapid recent growth, suggesting these nations are emerging contributors in the field.

Overall, co-authorship patterns by country position North America and Europe as the dominant centers of Alzheimer’s RCT research. At the same time, Asian and Middle Eastern nations are emerging as growing contributors.

## DISCUSSION

This bibliometric analysis provides a comprehensive overview of the landscape of RCTs in AD over the past 15 years, highlighting major research themes, temporal trends, and international collaboration patterns. The findings underscore a clear paradigm shift from pharmacological monotherapy approaches toward multidomain and precision-based interventions, while also emphasizing the increasing role of global collaboration and digital innovation in shaping the future of AD research.

Historically, RCTs in AD primarily evaluated symptomatic therapies such as cholinesterase inhibitors (donepezil, rivastigmine, galantamine) and memantine, which provide modest benefits in cognition and daily function but do not alter disease progression^15,16^. Consistent with our results, studies conducted between 2010 and 2015 were largely focused on these pharmacological targets, with amyloid-β (Aβ) remaining a dominant theme. This emphasis reflects the amyloid cascade hypothesis, which has long provided the central framework for therapeutic development in AD^17^. However, since 2015, there has been a notable increase in RCTs investigating monoclonal antibodies targeting Aβ and tau, such as aducanumab, donanemab, and lecanemab^18,19,20^. Although these therapies have generated significant debate regarding efficacy, safety, and clinical meaningfulness, they mark a turning point in the field by demonstrating the feasibility of disease-modifying interventions. Their prominence in recent bibliometric networks suggests that pharmacological innovation remains a cornerstone of AD RCTs, albeit with shifting priorities toward biologically targeted therapies. Importantly, these agents have reinvigorated research into biomarkers and imaging, as trial designs increasingly incorporate amyloid and tau positron emission tomography (PET) and cerebrospinal fluid (CSF) measures to enrich study populations and track outcomes^21,22^.

In parallel, our keyword co-occurrence analysis revealed the growing influence of lifestyle and prevention-based RCTs, particularly after 2015. Landmark studies such as the Finnish Geriatric Intervention Study to Prevent Cognitive Impairment and Disability (FINGER) and its international extensions (e.g., U.S. POINTER, J-MINT in Japan) have demonstrated that multidomain interventions targeting diet, exercise, cognitive training, and vascular risk factors can improve cognition or slow decline among at-risk older adults^23,24^.These trials have redefined the scope of AD research by integrating modifiable risk factors into prevention strategies, consistent with global public health initiatives emphasizing brain health promotion across the life course^25^.

The prominence of lifestyle-related clusters in our analysis indicates that non-pharmacological interventions are now firmly established as a complementary research frontier. Notably, these approaches are particularly relevant in light of the limited efficacy of current pharmacological options and the increasing global prevalence of AD. Furthermore, their scalability and applicability across diverse populations make them critical for reducing disease burden in both high- and low-resource settings^26^.

Our results also highlighted emerging clusters centered on gut microbiome research, digital health technologies, and machine learning applications. The role of systemic inflammation and gut–brain axis in AD pathophysiology has gained momentum, with early RCTs investigating probiotic supplementation and dietary interventions as modulators of cognition and biomarker profiles^27,28^, digital health innovations, including smartphone-based cognitive training, wearable activity trackers, and telehealth interventions, have become increasingly relevant in the post-COVID-19 era, enabling remote participation and real-time monitoring^29^.

Machine learning and artificial intelligence applications in AD trials are rapidly expanding, ranging from patient stratification and early diagnosis to adaptive trial designs and outcome prediction^30^.These domains are relatively nascent but align with the broader shift toward precision medicine in neurodegenerative diseases. Their presence in bibliometric mapping suggests that the next generation of AD RCTs may integrate computational tools with traditional methodologies to enhance efficiency and personalization.

Co-authorship analyses confirmed that North America and Europe remain the dominant hubs of AD RCT research, consistent with their established infrastructure, funding capacity, and long-standing academic–industry partnerships. The United States, in particular, not only contributed the largest volume of publications but also demonstrated the highest citation impact, underscoring its central role in shaping global therapeutic strategies.

European countries such as Finland, Sweden, and the Netherlands were strongly represented in lifestyle and prevention-focused trials, reflecting their leadership in multidomain intervention research (e.g., FINGER and its derivatives)^7^. In Asia, rapid growth in China, Japan, and South Korea reflects increasing national investment in neurodegenerative disease research, as well as demographic pressures from rapidly aging populations^31^. Singapore’s exceptionally high normalized citation impact, averaging over 90 citations per paper, highlights its influential role despite smaller output, likely attributable to participation in multinational trials and emphasis on high-quality translational research^32^. Our findings also revealed a cluster of emerging contributors, including Iran, India, Turkey, and Brazil, where research output has expanded rapidly since 2020. Although these countries currently contribute fewer RCTs, their trajectory suggests growing engagement with global networks, which may diversify study populations and improve the generalizability of trial findings.

The identification of “clinical trial methodology” as a distinct keyword cluster reflects the central role of study design in shaping the quality and credibility of evidence. Terms such as “double-blind,” “placebo,” and “multicenter” highlight the persistent emphasis on methodological rigor, which is essential in a field where outcomes are often heterogeneous and influenced by numerous biological and psychosocial factors. The growing incorporation of biomarkers, stratification strategies, and adaptive trial designs demonstrates that RCT methodology in AD has evolved to address prior shortcomings, such as high attrition rates, lack of reproducibility, and limited external validity^33^.

Bibliometric mapping adds value by providing a macro-level perspective on these methodological trends, complementing systematic reviews and meta-analyses that typically focus on specific interventions or outcomes. While conventional reviews synthesize efficacy data, bibliometric analyses highlight how research priorities and strategies shift over time, thereby offering a broader lens for understanding the dynamics of the field. This is particularly relevant in AD, where failure rates of pharmacological RCTs have historically been high, and where novel methodological approaches are crucial for advancing therapeutic development^34^.

Our results confirm a paradigm shift in AD research, with increasing emphasis on multimodal and precision-driven interventions. This transition reflects the growing consensus that no single therapeutic strategy is likely to be sufficient in addressing the multifactorial nature of AD pathophysiology. Integrating pharmacological agents with lifestyle modifications and supportive behavioral strategies aligns with emerging models of chronic disease management, which prioritize holistic and patient-centered care^35^.

Moreover, the prominence of digital health and machine learning within recent bibliometric clusters suggests that future RCTs will increasingly leverage technology to enhance efficiency, reduce costs, and improve participant diversity. These innovations may also mitigate long-standing challenges in AD trials, including recruitment barriers, underrepresentation of minority populations, and the logistical demands of in-person assessments^36,37^.

A major strength of this study is the comprehensive two-stage search strategy that maximized dataset coverage by combining PubMed’s clinical specificity with WoS’s enriched metadata. This approach ensured robust identification of RCTs while allowing for advanced bibliometric analyses, including keyword co-occurrence, co-authorship, and citation impact. By restricting the timeframe to the past 15 years, we captured contemporary trends while maintaining sufficient historical context to detect paradigm shifts.

The application of VOSviewer provided a detailed visualization of relationships among keywords, authors, and countries, facilitating the identification of distinct research clusters and emerging domains. Furthermore, by integrating temporal analyses, we were able to demonstrate how the focus of AD RCTs has evolved, providing valuable insights for stakeholders in academia, clinical practice, and policy.

Several limitations must be acknowledged. First, bibliometric analyses are inherently dependent on database coverage and indexing practices. Although the combination of PubMed and WoS ensured broad coverage, certain RCTs published in non-indexed journals or in languages other than English may have been excluded. Second, bibliometric indicators such as citation counts are influenced by factors beyond scientific merit, including journal visibility, open-access status, and citation practices within subfields^38^. As a result, highly cited studies may not necessarily reflect the most clinically impactful findings.

Third, while keyword co-occurrence provides valuable insights into thematic structures, it is influenced by author-selected terminology, which may vary across contexts. The clustering algorithm itself is also sensitive to parameter choices, and while we employed widely accepted thresholds and normalization methods, some degree of subjectivity in interpretation is unavoidable. Finally, this study did not evaluate trial quality or clinical outcomes, as bibliometric approaches cannot substitute for critical appraisal of individual studies.

The findings of this bibliometric analysis highlight several implications for the future of AD research. First, the continued pursuit of disease-modifying pharmacological therapies remains critical, particularly as monoclonal antibodies targeting amyloid and tau advance toward regulatory approval and clinical implementation. However, the limited and variable efficacy of these agents underscores the necessity of combining pharmacological and non-pharmacological strategies. Future RCTs should explore integrative models that incorporate pharmacotherapy, vascular risk management, cognitive training, and psychosocial support.

Second, the rise of lifestyle and prevention-focused RCTs suggests that earlier intervention may be more effective in delaying or preventing cognitive decline than treatments initiated in symptomatic stages. Large-scale, multinational studies are needed to validate these approaches in diverse populations, particularly in low- and middle-income countries where AD prevalence is rapidly increasing^39^. Third, digital health and machine learning tools should be integrated into RCT design to optimize recruitment, monitoring, and outcome prediction, while also enhancing inclusivity and cost-effectiveness^40^. Finally, as emerging economies increase their contributions to AD RCTs, fostering international collaborations and capacity-building initiatives will be essential for ensuring globally relevant and equitable advances in treatment.

In conclusion, this bibliometric analysis provides a comprehensive overview of RCTs in AD published over the past 15 years. The findings demonstrate a clear transition from traditional pharmacological strategies, primarily focused on cholinesterase inhibitors and amyloid-targeting approaches, toward multidomain interventions that incorporate lifestyle modification, cognitive training, and prevention-oriented strategies. In parallel, advances in biomarker-guided trial design and the emergence of digital health and machine learning applications highlight the field’s progression toward precision medicine.

Geographic and collaborative analyses identified North America and Europe as the dominant centers of research, while Asian and Middle Eastern countries are emerging as active contributors with growing impact. This diversification of global participation is likely to enhance the generalizability and applicability of future trial findings. Despite methodological advances, persistent challenges remain, including limited efficacy of pharmacological therapies, high failure rates of disease-modifying agents, and the underrepresentation of diverse populations in clinical trials.

In conclusion, these findings demonstrate a paradigm shift in AD RCTs from traditional pharmacological strategies toward multidomain, precision-driven interventions, with digital innovations emerging as likely frontiers of research. Strengthening global collaboration, advancing methodological innovation, and prioritizing prevention across diverse populations will be essential for reducing the burden of AD worldwide.

## Data Availability

No new data were generated or analyzed in this study.

## References

[1] World Health Organization (2025). Dementia: Fact sheet [Internet].

[2] Alzheimer’s Disease International (2018). World Alzheimer Report 2018: the state of the art of dementia research: new frontiers.

[3] Schulz KF, Grimes DA (2002). Generation of allocation sequences in randomised trials: chance, not choice. Lancet.

[4] Cummings J, Osse AML, Cammann D, Powell J, Chen J (2024). Anti-amyloid monoclonal antibodies for the treatment of Alzheimer’s Disease. BioDrugs.

[5] Chundu UC, Thiriveedhi SR, Bhatti C, Mupparaju JS, Otinashvili N, Gelishvili E (2025). A systematic review of the efficacy and safety of anti-amyloid monoclonal antibodies in Alzheimer’s Disease. Cureus.

[6] Raina P, Santaguida P, Ismaila A, Patterson C, Cowan D, Levine M (2008). Effectiveness of cholinesterase inhibitors and memantine for treating dementia: evidence review for a clinical practice guideline. Ann Intern Med.

[7] Ngandu T, Lehtisalo J, Solomon A, Levälahti E, Ahtiluoto S, Antikainen R (2015). A 2 year multidomain intervention of diet, exercise, cognitive training, and vascular risk monitoring versus control to prevent cognitive decline in at-risk elderly people (FINGER): a randomised controlled trial. Lancet.

[8] Rosenberg A, Mangialasche F, Ngandu T, Solomon A, Kivipelto M (2020). Multidomain interventions to prevent cognitive impairment, Alzheimer’s Disease, and dementia: from FINGER to World-Wide FINGERS. J Prev Alzheimers Dis.

[9] Donthu N, Kumar S, Mukherjee D, Pandey N, Lim WM (2021). How to conduct a bibliometric analysis: An overview and guidelines. J Bus Res.

[10] Chen C (2006). CiteSpace II: Detecting and visualizing emerging trends and transient patterns in scientific literature. J Am Soc Inf Sci Technol.

[11] Mc Laughlin J, Scotton WJ, Ryan NS, Hardy JA, Shoai M (2024). Assessing clinical progression measures in Alzheimer’s disease trials: A systematic review and meta-analysis. Alzheimers Dement.

[12] Pucci IM, Aguiar AF, Pucci RM, Casonatto J, Borghi SM (2024). Systematic review and meta-analysis of randomized controlled trials on the effects of exercise interventions on amyloid beta levels in humans. Exp Brain Res.

[13] Zeng B, Tang C, Wang J, Yang Q, Ren Q, Liu X (2024). Pharmacologic and nutritional interventions for early Alzheimer’s Disease: a systematic review and network meta-analysis of randomized controlled trials. J Alzheimers Dis.

[14] Dong X, Yan L, Huang L, Guan X, Dong C, Tao H (2018). Repetitive transcranial magnetic stimulation for the treatment of Alzheimer’s disease: A systematic review and meta-analysis of randomized controlled trials. PLoS One.

[15] Tricco AC, Soobiah C, Berliner S, Ho JM, Ng CH, Ashoor HM (2013). Efficacy and safety of cognitive enhancers for patients with mild cognitive impairment: a systematic review and meta-analysis. CMAJ.

[16] Birks JS, Chong LY, Grimley Evans J (2015). Rivastigmine for Alzheimer’s disease. Cochrane Database Syst Rev.

[17] Hardy J, Selkoe DJ (2002). The amyloid hypothesis of Alzheimer’s disease: progress and problems on the road to therapeutics. Science.

[18] Sevigny J, Chiao P, Bussière T, Weinreb PH, Williams L, Maier M (2016). The antibody aducanumab reduces Aβ plaques in Alzheimer’s disease. Nature.

[19] Mintun MA, Lo AC, Evans CD, Wessels AM, Ardayfio PA, Andersen SW (2021). Donanemab in early Alzheimer’s Disease. N Engl J Med.

[20] van Dyck CH, Swanson CJ, Aisen P, Bateman RJ, Chen C, Gee M (2023). Lecanemab in early Alzheimer’s Disease. N Engl J Med.

[21] Jack CR, Bennett DA, Blennow K, Carrillo MC, Dunn B, Haeberlein SB (2018). NIA-AA Research Framework: Toward a biological definition of Alzheimer’s disease. Alzheimers Dement.

[22] Sperling RA, Rentz DM, Johnson KA, Karlawish J, Donohue M, Salmon DP (2014). The A4 study: stopping AD before symptoms begin?. Sci Transl Med.

[23] Rosenberg A, Ngandu T, Rusanen M, Antikainen R, Bäckman L, Havulinna S (2018). Multidomain lifestyle intervention benefits a large elderly population at risk for cognitive decline and dementia regardless of baseline characteristics: The FINGER trial. Alzheimers Dement.

[24] Sugimoto T, Sakurai T, Akatsu H, Doi T, Fujiwara Y, Hirakawa A (2021). The Japan-Multimodal Intervention Trial for Prevention of Dementia (J-MINT): the study protocol for an 18-month, multicenter, randomized, controlled trial. J Prev Alzheimers Dis.

[25] Livingston G, Huntley J, Sommerlad A, Ames D, Ballard C, Banerjee S (2020). Dementia prevention, intervention, and care: 2020 report of the Lancet Commission. Lancet.

[26] Norton S, Matthews FE, Barnes DE, Yaffe K, Brayne C (2014). Potential for primary prevention of Alzheimer’s disease: an analysis of population-based data. Lancet Neurol.

[27] Kobayashi Y, Kuhara T, Oki M, Xiao JZ (2019). Effects of Bifidobacterium breve A1 on the cognitive function of older adults with memory complaints: a randomised, double-blind, placebo-controlled trial. Benef Microbes.

[28] Jiang C, Li G, Huang P, Liu Z, Zhao B (2017). The gut microbiota and Alzheimer’s Disease. J Alzheimers Dis.

[29] Rentz DM, Dekhtyar M, Sherman J, Burnham S, Blacker D, Aghjayan SL (2016). The feasibility of at-home iPad cognitive testing for use in clinical trials. J Prev Alzheimers Dis.

[30] Ding Y, Sohn JH, Kawczynski MG, Trivedi H, Harnish R, Jenkins NW (2019). A deep learning model to predict a diagnosis of Alzheimer Disease by using (18)F-FDG PET of the brain. Radiology.

[31] Jia J, Wang F, Wei C, Zhou A, Jia X, Li F (2014). The prevalence of dementia in urban and rural areas of China. Alzheimers Dement.

[32] Ho Y-S (2014). A bibliometric analysis of highly cited articles in materials science. Curr Sci.

[33] Winblad B, Amouyel P, Andrieu S, Ballard C, Brayne C, Brodaty H (2016). Defeating Alzheimer’s disease and other dementias: a priority for European science and society. Lancet Neurol.

[34] Cummings JL, Morstorf T, Zhong K (2014). Alzheimer’s disease drug-development pipeline: few candidates, frequent failures. Alzheimers Res Ther.

[35] Canevelli M, Cesari M (2017). Cognitive frailty: far from clinical and research adoption. J Am Med Dir Assoc.

[36] Watson JL, Ryan L, Silverberg N, Cahan V, Bernard MA (2014). Obstacles and opportunities in Alzheimer’s clinical trial recruitment. Health Aff (Millwood).

[37] Grill JD, Karlawish J (2010). Addressing the challenges to successful recruitment and retention in Alzheimer’s disease clinical trials. Alzheimers Res Ther.

[38] Bornmann L, Leydesdorff L (2016). Skewness of citation impact data and covariates of citation distributions: A large-scale empirical analysis based on Web of Science data. J Informetr.

[39] Prince M, Wimo A, Guerchet M, Ali G-C, Wu Y-T, Prina M (2015). World Alzheimer Report 2015. The global impact of dementia: an analysis of prevalence, incidence, cost and trends. Alzheimer’s Disease International.

[40] Berron D, Olsson E, Andersson F, Janelidze S, Tideman P, Düzel E (2024). Remote and unsupervised digital memory assessments can reliably detect cognitive impairment in Alzheimer’s disease. Alzheimers Dement.

